# Use of portable air purifiers to reduce aerosols in hospital settings and cut down the clinical backlog: a critical analysis

**DOI:** 10.1017/S0950268823000535

**Published:** 2023-05-19

**Authors:** Robert Malek, Usmaan Al-Shehab, David F. Lo

**Affiliations:** 1 Department of Medicine, Rowan University School of Osteopathic Medicine, Stratford, NJ, USA; 2 American Preventive Screening & Education Association (APSEA), Stratford, NJ, USA; 3 Department of Biology, Rutgers, The State University of New Jersey, New Brunswick, NJ, USA

Reading the article titled ‘Use of portable air purifiers to reduce aerosols in hospital settings and cut down the clinical backlog’ by Jacob Salmonsmith et al. [1] encouraged us to publish a commentary on the extrapolated conclusions regarding the use of portable air cleaners to reduce the concentration of aerosols in patient environments. We hope these perspectives may provide insight into areas that may require further research and improvement.

In the aforementioned article, the authors summarised that the onset of the Covid-19 virus saw a substantial increase in the patient waiting lists for doctor consultations and procedures in healthcare settings in order to decrease disease transmission [1]. The researchers proposed that portable air cleaners (PACs) can be a cost-effective alternative to built-in mechanical ventilation systems to reduce potentially infectious aerosol concentrations. The experiment found that the use of PACs significantly reduced aerosol concentrations, which may decrease the risk of disease transmission and potentially lead to a reduction in clinical waiting lists [1].

In order to mimic the flow of aerosols out of someone’s mouth, saline was dispensed via a nebuliser into a room and monitored with a laser spectrometer (aerodynamic particle sizer (APS)) [1]. Since the start of the Covid-19 pandemic, mask-wearing mandates have been enforced at varying levels, including federal, state, and independent businesses. Studies have shown that aerosol spread can be reduced by up to 60% depending on the type of mask worn [2]. None of the simulations run in this experiment included alterations to the nebuliser system that would mimic the physical restrictions brought about by mask-wearing in clinical settings. The degree of aerosols released into the environment and the dynamics of aerosol outflow within worn masks may have led results to overstate the effectiveness of PACs in healthcare settings where mask-wearing may be mandated.

In the simulations that were run, only one nebuliser was placed in the room to mimic the outflow of aerosols from a patient [1]. In unsimulated healthcare settings, there may be a larger population of people conversing and releasing potentially infectious droplets while sitting at varying angles. This resulted in more unpredictability when metrics such as the amount of aerosol released and the interactions of multiple particle streams were measured in unsimulated settings as compared to simulated settings. To address this, the authors may consider conducting additional simulations with different quantities of nebulisers in each room.

In addition to the presence of only one nebuliser, there was also only one APS present to detect the produced aerosols [1]. The simulations depicted a circumstance in which only two people were present in a healthcare setting. The degree of aerosols detected by the single APS and the timeframe in which the particles were removed from the air may not accurately depict how infectious droplets spread to a group of people in immediate circumstances. In a real setting, there may be too many people present in a room and making contact with aerosols before PACs have time to remove them from the environment. This factor could potentially contribute to the ongoing issue of long waiting lists.

To summarise, mask-wearing as well as the number of nebulisers and APSs used during experimentation can be some additional aspects to consider when analysing how PACs may play a role in reducing aerosol spread from person to person in healthcare settings. By incorporating these factors into future research designs, a more accurate simulation of a healthcare setting can be created for testing. We look forward to reading about future studies that provide insight into these factors.

## Abbreviations

APS: aerodynamic particle sizer
PAC: portable air cleaner
